# Xanthogranulomatous Cholecystitis Masquerading as Gallbladder Cancer: Can It Be Diagnosed Preoperatively?

**DOI:** 10.1155/2014/253645

**Published:** 2014-10-27

**Authors:** Ashwin Rammohan, Sathya D. Cherukuri, Jeswanth Sathyanesan, Ravichandran Palaniappan, Manoharan Govindan

**Affiliations:** The Institute of Surgical Gastroenterology & Liver Transplantation, Centre for GI Bleed, Division of HPB Diseases, Stanley Medical College Hospital, Old Jail Road, Chennai 600 001, India

## Abstract

*Background*. Xanthogranulomatous cholecystitis (XGC) is often misdiagnosed as gallbladder cancer (GBC). We aimed to determine the preoperative characteristics that could potentially aid in an accurate diagnosis of XGC masquerading as GBC. *Methods*. An analysis of patients operated upon with a preoperative diagnosis of GBC between January 2008 and December 2012 was conducted to determine the clinical and radiological features which could assist in a preoperative diagnosis of XGC. *Results*. Out of 77 patients who underwent radical cholecystectomy, 16 were reported as XGC on final histopathology (Group A), while 60 were GBC (Group B). The incidences of abdominal pain, cholelithiasis, choledocholithiasis, and acute cholecystitis were significantly higher in Group A, while anorexia and weight loss were higher in Group B. On CT, diffuse gallbladder wall thickening, continuous mucosal line enhancement, and submucosal hypoattenuated nodules were significant findings in Group A. CT findings on retrospect revealed at least one of these findings in 68.7% of the cases. *Conclusion*. Differentiating XGC from GBC is difficult, and a definitive diagnosis still necessitates a histopathological examination. An accurate preoperative diagnosis requires an integrated review of clinical and characteristic radiological features, the presence of which may help avoid radical resection and avoidable morbidity in selected cases.

## 1. Introduction

Xanthogranulomatous cholecystitis (XGC) is an inflammatory disease of the gallbladder, characterised by a focal/diffuse destructive inflammatory process followed by marked proliferative fibrosis along with infiltration of macrophages and foamy cells [1]. Its incidence ranges from 0.7 to 10% [1–3]. XGC exhibits similar imaging and intraoperative findings as those of gallbladder cancer (GBC), leading to its frequent misdiagnosis [2–5]. Imaging does shed some light on this conundrum preoperatively, but diagnosis is often a postoperative histological surprise [6–12]. These lesions are easily confused with more sinister neoplasms, and hence there needs to be an increased awareness of this tumour mimic, particularly in endemic areas [6–12]. Identifying the preoperative differences between XGC and GBC is imperative, as it would help avert unnecessary morbidity especially in the form of radical surgery. This study aimed to determine the preoperative characteristics of XGC that could potentially aid in an accurate diagnosis of XGC masquerading as GBC.

## 2. Methods

**Figure 1:**
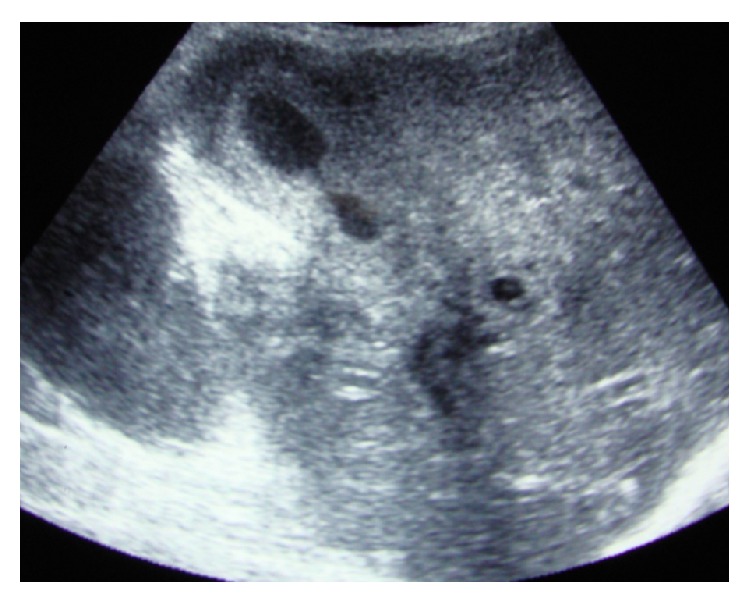
Ultrasound showing smooth uniform thickening of gallbladder wall.

**Figure 2:**
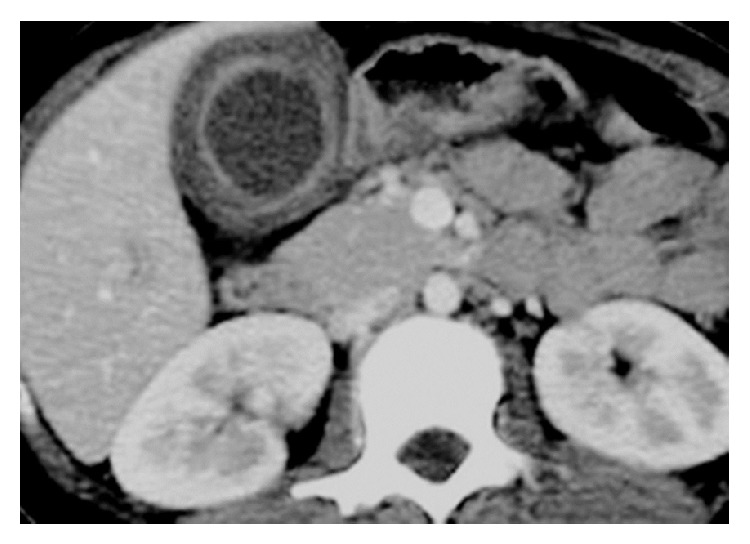
Contrast-enhanced CT showing smooth circumferential gallbladder wall thickening with a continuous contrast—enhanced mucosal line.

An analysis from a prospectively collected database of patients operated-upon in our department with a preoperative diagnosis of GBC between January 2008 and December 2012 was done. All patients who underwent a radical cholecystectomy were considered for the study. Out of a total of 77 patients, 76 were included in the study; one patient had GBC associated with XGC and was excluded from the analysis. Based on their final histopathology, the patients were divided into two groups. Out of the 76 patients, 16 were reported as XGC on final histopathology (Group A), while 60 were GBC (Group B). Data was collected with regard to clinical features, tumor markers (CEA, CA19.9), and radiological investigations {ultrasonogram (USG) and/or CT}. Particular stress was given on certain radiological features like the thickness of the gallbladder wall, patterns of wall thickening (focal versus diffuse), continuity of mucosal line (continuous versus disrupted), enhancement characteristics of mucosa (homogeneous versus heterogeneous), presence of submucosal hypoattenuated nodules or bands, and presence or absence of enlarged lymph nodes (Figures 1 and 2). Clinical and radiological features were compared between XGC and GBC to determine features which could assist in a preoperative diagnosis of XGC.

## 3. Statistical Analysis

Nominal variables were comparedusing the chi-square or Fisher's exact probability test, while continuous variables were compared using Student's *t*-test. A *P* value < 0.05 was considered statistically significant. All statistical analyses were performed using SPSS 20.0 statistical package (SPSS, Chicago, IL, USA).

## 4. Results

**Figure 3:**
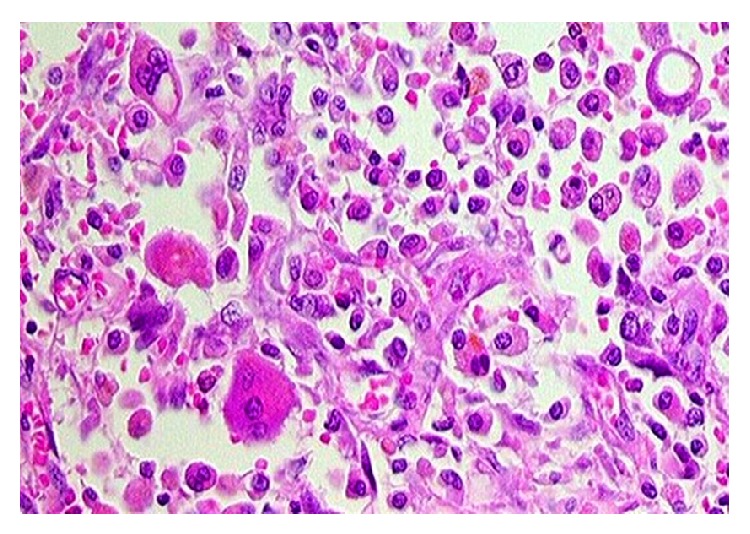
Histopathology slide of XGC showing foamy macrophages and giant cells in the wall of the gallbladder.

Out of 77 patients with resectable disease who underwent radical cholecystectomy, 16 were reported as XGC (Group A), while 60 patients had a histopathology of GBC (Group B) (Figure 3). One patient had GBC associated with XGC and was excluded from the study. Analysis of the clinical features revealed a higher incidence of abdominal pain and acute cholecystitis in Group A. Cholelithiasis and choledocholithiasis were also more commonly seen in Group A. The incidences of anorexia and weight loss were significantly higher in Group B. There were no significant differences with respect to age, gender, presence of jaundice, and the presence of a palpable mass. Tumour markers (serum CEA and CA19.9) were found to be significantly elevated in patients with GBC (Table 1).

On comparison of radiological features, patients with XGC were more frequently found to have a diffuse gallbladder wall thickening, continuous mucosal line enhancement, and submucosal hypoattenuated nodules or bands (Figures 1 and 2 and Table 2). There were no significant differences in the thickness of gallbladder wall or enlargement of lymph nodes between the groups. On retrospect, at least one of these findings was noted in 68.7% (11/16) cases with XGC.

## 5. Discussion

XGC can exhibit similar imaging and intraoperative findings as those of GBC and are easily misdiagnosed, often leading to unnecessary radical surgery [1, 4, 13]. An increased awareness combined with an increased accuracy of preoperative and intraoperative diagnosis and an algorithmic approach to XGC could help avoid extended resections. Analogous to the results of a Chinese study, our data suggests that the presence of abdominal pain, acute cholecystitis, choledocholithiasis, and cholelithiasis may portend a diagnosis of XGC [6]. The role of tumour markers remains unresolved, with some studies showing no significant correlations with diagnosis [2, 3, 5, 6]. In our study, raised levels of tumour markers correlated well with the incidence of GBC and could be used in the differentiation of XGC from GBC.

Extravasation of bile into the gallbladder wall with involvement of Rokitansky-Aschoff sinuses is a potential precipitating factor for XGC [6–12]. These lead to formation of submucosal abscesses or xanthogranulomas, which show up on CECT as submucosal hypoattenuated nodules occupying large areas of the gallbladder wall, a sign highly suggestive of XGC. Other features like the continuous mucosal line in a thickened gallbladder wall and the presence of gallstones in a background of chronic gallbladder disease have been reported as being highly suggestive of XGC [6–12]. The radiological findings indicative of XGC in our series concurred with those reported in literature.

EUS-guided FNA (EUS-FNA) is a useful modality for sampling various targets. Even though EUS-FNA is a feasible and safe method for obtaining samples, its role in the diagnostic workup of gallbladder lesions remains undefined. While a positive FNAC confirms the diagnosis of GBC, a negative sample does not shed much light. The overall sampling adequacy is reported to be 86%. The accuracy of EUS-FNA for detecting malignancy and for the final diagnosis is approximately 93% and 80%, respectively [13, 14]. Sampling errors in the form of samples from nonrepresentative areas along with a confounding factor of coexistence of XGC and GBC limit the widespread applicability of EUS-FNA in XGC [13, 14]. Intraoperative frozen section examination is an efficient method for exclusion of GBC. Its liberal use also helps rule out the simultaneous occurrence of GBC/XGC, thereby guiding optimum surgery [2, 6, 15–17]. Frozen section in combination with immunohistochemistry has shown to be highly sensitive in their ability to differentiate XGC from GBC [18].

Controversy exists regarding the use of laparoscopic cholecystectomy (LC) in patients with XGC [19–21]. The intense chronic inflammatory process can make the procedure arduous and hazardous, and hence in any patient with a difficult laparoscopic cholecystectomy, an on-table differential diagnosis of XGC must be entertained amongst others. Multiple series have attested to the safety of LC in XGC, with no increase in the morbidity as compared to an open procedure. There is indeed a higher incidence of conversion to an open procedure, but this low threshold for conversion to open surgery enables a better assessment of the lesion and results in superior outcomes with regard to mortality and morbidity [1, 3, 19–21].

**Figure 4:**
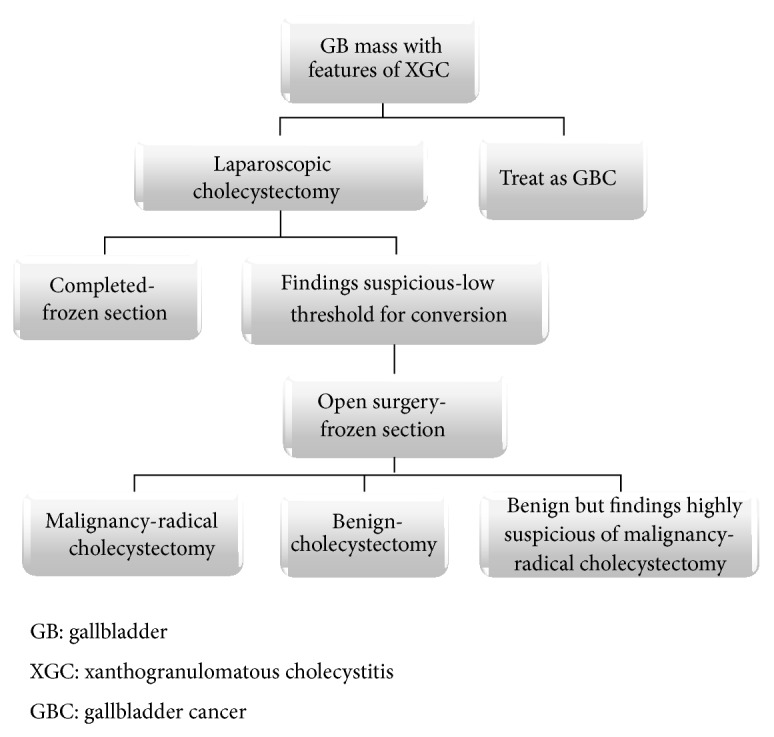
Algorithm for management of gallbladder mass with features of XGC.

A combination of clinical, radiological factors combined with a liberal application of intraoperative frozen section examination can aid in the diagnosis and surgery for XGC. To help guide the surgeon towards a structured and rationalized management of XGC, based on our study, a simple algorithm has been proposed (Figure 4). As suggested in our algorithm, despite all radiological and frozen section analyses, a high index of suspicion of GBC on the part of the operating surgeon warrants a radical surgery.

## 6. Conclusion

Differentiating XGC from GBC is a diagnostic conundrum. Making this distinction preoperatively or intraoperatively is difficult, and a definitive diagnosis still necessitates a histopathological examination. An accurate preoperative diagnosis requires an integrated review of clinical and characteristic radiological features, the presence of which may help avoid radical resection and avoidable morbidity in selected cases.

## Figures and Tables

**Table 1:** Comparison of clinical features and tumour markers between XGC and GBC.

	XGC	GBC	*P* value
Age (mean ± SD)	56.2 ± 12.4	58 ± 11.1	NS
Male/female	6/9	25/35	NS
Abdominal pain (%)	16 (100)	37 (61)	*P* < 0.01
Acute cholecystitis (%)	12 (75)	9 (15)	*P* < 0.01
Cholelithiasis (%)	11 (68.8)	17 (28.3)	*P* < 0.01
Choledocholithiasis (%)	4 (25)	2 (3.3)	*P* < 0.01
Loss of weight (%)	3 (18.8)	36 (60)	*P* < 0.01
Loss of appetite (%)	9 (56)	42 (70)	*P* < 0.05
Diabetes (%)	5 (31.3)	18 (30)	NS
Jaundice	2 (12.5)	8 (13.3)	NS
Palpable mass	5 (18.8)	7 (11.7)	NS
Tumour markers			
CEA (≥4 ng/mL)	0	49	*P* < 0.01
CA19.9 (≥20 IU/mL)	2	41	*P* < 0.01

**Table 2:** Comparison of radiological findings between XGC and GBC.

Radiological findings	XGC	GBC	*P* value
GB wall thickness (mean ± SD, mm)	14.1 ± 4.9	13.6 ± 6.1	NS
Diffuse GB wall thickening	4 (36.6%)	4 (6.6%)	*P* < 0.01
Continuous mucosal line	8 (50%)	6 (10%)	*P* < 0.01
Submucosal hypoattenuated nodules/band	9 (56.2%)	10 (16.7%)	*P* < 0.01
Lymph node enlargement	10 (62.5%)	53 (88.3%)	NS
